# Smoking, Cognitive Function and Mortality in a U.S. National Cohort Study

**DOI:** 10.3390/ijerph8093628

**Published:** 2011-09-07

**Authors:** Richard F. Gillum, John Kwagyan, Thomas O. Obisesan

**Affiliations:** 1 Geriatrics Division, Department of Medicine, Howard University, 2041 Georgia Ave NW, Washington, DC 20060, USA; E-Mail: tobisesan@howard.edu; 2 Georgetown-Howard Universities Center for Clinical and Translational Science, 2041 Georgia Ave NW, Washington, DC 20060, USA; E-Mail: jkwagyan@howard.edu

**Keywords:** smoking, memory, mortality, cotinine

## Abstract

Previous studies report that low levels cognitive function and history of smoking are associated with increased mortality risk. Elderly smokers may have increased risk of dementia, but risk in former smokers is unclear. We tested the hypotheses that the harmful effect of impaired cognitive function as related to mortality is greater in persons smoking at baseline than in others. Further, we used serum cotinine levels to assess recall bias of smoking history by cognitive function level. Data were analyzed from a longitudinal mortality follow-up study of 4,916 American men and women aged 60 years and over, examined in 1988–1994 with complete data followed an average 8.5 years. Measurements at baseline included smoking history, a short index of cognitive function (SICF), serum cotinine and socio-demographics. Death during follow-up occurred in 1,919 persons. In proportional hazards regression analysis, a significant interaction of current smoking with cognitive function was not found; but there was a significant age-smoking interaction. After adjusting for confounding by age or multiple variables, current smoking associated with over 2-fold increased mortality (hazards ratio and 95% confidence limits current versus never smoking 2.13, 1.75–2.59) and SICF with 32% reduction in mortality; top versus bottom SICF stratum 0.68, 0.53–0.88). Serum cotinine data revealed substantial recall bias of smoking history in persons with cognitive impairment. However analyses correcting for this bias did not alter the main conclusions: In a nationwide cohort of older Americans, analyses demonstrated a lower risk of death independent of confounders among those with high SICF scores and never smokers, without a significant interaction of the two.

## 1. Introduction

Both history of smoking and impaired cognitive function are prevalent concomitants of aging in industrialized nations [1,2]. Cohort studies of current smoking in elderly persons and dementia or Alzheimer’s disease suggest a positive association [3,4]. Validity of smoking history in persons with varying degrees of cognitive impairment could be a source of bias in such studies. Cognitive function has been found to predict subsequent mortality in elderly adults in a number of previous studies [5]. Mechanisms remain obscure. Smoking has been found to be positively related to increased mortality from multiple causes [1]. Mechanisms may include inflammation and oxidative stress leading to atherosclerosis and carcinogenesis. However, information regarding interactions of cognitive function and smoking are lacking in studies of mortality.

We test the hypothesis that the effect of cognitive function score on mortality is modified by smoking, the effect being less among never smokers compared to smokers. We further test the hypothesis that the validity of smoking history is directly associated with cognitive function level. We analyzed available data from a national health examination survey linked mortality file conducted with scientific sampling and state-of-the-art interviewing, examination and laboratory methods.

## 2. Methods

The Third National Health and Nutrition Examination Survey (NHANES III) was conducted in 1988–1994 on a nationwide multi-stage probability sample of 39,695 persons from the civilian, non-institutionalized population aged 2 months and over of the United States [6]. Persons aged 60 and over, African Americans, and Mexican Americans were over-sampled. Details of the plan, sampling, operation, response and institutional review board approval have been published as have procedures used to obtain informed consent and to maintain confidentiality of information obtained [6]. The personal interviews and physical and laboratory examinations of NHANES III subjects provided the baseline data for the study. This analysis was based on the public-use version of the NHANES III linked-mortality file with mortality data through 2000. Of 33,994 persons with baseline interview data, 13,944 were under age 17 and 26 lacked data for matching leaving 20,024 eligible for mortality follow-up. The NHANES III linked mortality file contains information based upon the results from a probabilistic match between NHANES III and the NCHS National Death Index records. The NHANES III linked mortality file provides mortality follow-up data from the date of NHANES III survey participation (1988–1994) through 31 December 2000. Of the 20,022 interviewed persons with mortality follow-up, 6,588 were aged 60 years and over and eligible to have cognitive function testing performed, 6,339 of whom had valid cognitive function data. After excluding persons with missing data for any of the variables shown in the tables, 4,916 persons aged 60 and over with complete data remained for mortality analyses (1,919 deaths). The length of follow-up of survivors averaged 8.5 years.

Questions assessing mental cognition were asked only of respondents aged 60 years or older and not to proxy respondents [7]. These questionnaires were designed for administration in a bilingual (English/Spanish) format so that respondents could be interviewed in their preferred language. The neuropsychological measures used in the NHANES III study, were selected to assess cognitive functions typically affected in dementia. A short cognitive function index (SICF) was constructed for this analysis from these items administered at home interview and/or at a Mobile Examination Center to assess orientation, recall and attention (Appendix 1) [7]. Cronbach’s alpha for the index was 0.82 (n = 4916). Participants were asked, “Have you smoked at least 100 cigarettes in your entire life?” (If yes) “Do you smoke cigarettes now?” (If yes) “About how many cigarettes do you smoke per day?” Responses were used to classify persons as current, former and never smokers. Serum cotinine levels were measured by high-performance liquid chromatography and atmospheric-pressure chemical ionization tandem mass spectrometry [8–10]. Previous researchers have used the serum cotinine value of <14 ng/mL to identify non-smokers. Serum cotinine is directly proportional to absorbed nicotine and has a half-life of approximately 20 hours [8]. It is considered a better marker of smoking status than self-reported tobacco use. It has been used in a number of reports to document underreporting of smoking in various groups [8]. Cotinine data were available for 2,415 of the 4,916 persons for the validity sub-analysis. Compared to persons in the analysis (Table 1, Appendix 2), persons aged 60 and greater excluded were older (mean age 72.65 years) and more likely to be female (62%), black (10.5%), and not married (47%) but did not differ in Mexican ethnicity or region of residence.

Estimates of the risk of death from Cox proportional hazards regression models with time-to-event as the time scale and other statistics were computed using the survey weights and survey analysis procedures in Stata release 11. Survivors were censored at the date of the end of mortality follow-up. Proportional hazards assumptions were assessed by graphical methods. Logistic regression models were utilized to further examine the effect of smoking and SICF on the odds of death.

## 3. Results

### 3.1. Baseline Analysis

Table 1 and Appendix 2 show demographic characteristics, SICF score and biomedical variables by smoking status. Bivariate analyses revealed significant associations between smoking and death (p < 0.01), SICF score stratum and death (p < 0.01) and between stratum of SICF score and smoking status (p = 0.03). In multivariate linear regression analyses, neither baseline SICF scores of current nor former smokers differed significantly from those of never smokers at baseline after adjusting for age or age category, gender, ethnicity, education and marital status (not shown). Among smokers, number of cigarettes smoked daily was not significantly associated with SICF score after adjusting for age or multiple confounders.

### 3.2. Follow-Up Analyses

In proportional hazards regression models, no significant interaction of SICF and smoking status was found (p = 0.119) with hazard of death. Results showed significant interaction of smoking with age (p = 0.002). Therefore, further analyses were done within age strata (Table 2). At age 60–69, both SICF score and smoking status were significant predictors of death over the follow-up period. However, the association for smoking decreased with advancing age becoming non-significant at 80+ years, unlike that for SICF score. Adjusting in addition for self-reported health status at baseline, yielded essentially similar results. Regression analyses were repeated using multiple logistic regression (Table 3). These yielded essentially similar results to those shown in Table 2.

### 3.3. Cognitive Function and Recall Bias

In past studies of younger populations, self-reported history of never and former smoking with serum cotinine ≥ 14 ng/mL were considered false negative histories. In this study, 97.7% (95% CI 5.8–15.4) of persons who reported current smoking had serum cotinine at that level. Results for those reporting former and never smoking are shown in Table 4. There was evidence for a higher rate of false negatives in those with low SICF scores than in those with high SICF scores, significantly so for those reporting never smoking.

To assess the effect of misclassification of smoking exposure on associations with mortality, the logistic models were repeated using serum cotinine level instead of smoking as the exposure variable (reference group: undetectable, medium: detectable but <14 ng/mL, high: ≥14 ng/mL). Adjusted odds ratios for the high group were somewhat lower than for the category current smokers in Table 3: all 2.30 (1.77–3.01, 60–69 2.99 (2.05–4.34), 70–79 2.05 (1.38–3.05), 80+ 1.03 (0.56–1.90). Adjusted odds ratios for SICF score were little changed and again there was no significant interaction of cotinine and SICF score.

## 4. Discussion

This analysis of data from the NHANES III linked-mortality file, a nation-wide representative sample, is the first study to test for interaction of cognitive function and smoking as predictors of survival in older Americans and the first to assess the effect of recall bias with low cognitive function on such analyses. Both smoking and poor cognitive function were found to independently predict diminished survival chances over follow-up, consistent with previous studies [5,11,12]. However, no interaction of the two was detected. A bivariate association between smoking and cognitive function was no longer significant after controlling for age or multiple confounders. Recall bias was substantial but correcting for this bias did not alter the main conclusions of the follow-up analysis. These findings support efforts to effect smoking cessation and to develop effective prevention of cognitive decline among the elderly.

The NHANES III provides population-based data on the independent association of smoking, cognitive function with survival in a nation-wide representative sample of Americans. Over-sampling of persons aged 60 and over permitted reliable estimates for this group [6]. However, several unavoidable limitations of the present study include possible bias arising from survey non-response and exclusion due to missing values for some variables, and from possible changes in smoking and cognitive function and/or other variables over the follow-up period. In Cox regression analysis, plots of -log-log survival curves suggested possible violations of proportional hazards assumptions. However, logistic regression analyses produced essentially the same results. A model including presence of major comorbidities at baseline produced essentially the same results as reported above (Table 2). However, the possibility of residual confounding by unmeasured variables cannot be excluded. Further longitudinal studies with repeated measures of smoking status, duration and amount, and multiple dimensions of cognition and Alzheimer’s disease biomarkers would be helpful.

## 5. Conclusions

In a nationwide cohort of older Americans, analyses demonstrated a lower risk of death independent of confounders among those with high SICF scores and never smokers without a significant interaction of the two.

## Figures and Tables

**Table 1:** Prevalence (%) of selected characteristics by smoking status at baseline in persons aged 60 years and over (N = 4,916).

	Smoking status			
	All	Never	Former	Current
**N**	4,916	2,336	1,827	753
**Dead**	32	27	34	39
**Cognitive function index**				
**<12**	19	10	17	23
**12–13**	37	35	38	37
**14–16**	19	21	19	14
**17**	25	23	26	26
**Female**	57	73	40	53
**Age 80+**	14	20	11	5
**Mexican American**	2	2	2	2
**African American**	8	6	6	11
**South region**	31	31	28	35
**Metropolitan residence**	44	43	46	40
**Not married**	40	46	31	44
**Education < 12 year**	41	41	38	45
**Fair-poor health**	28	26	29	32
**≥ 1 chronic illness**	56	52	62	52
**Mobility limitation**	33	34	30	37
**Low social support**	12	10	12	15
**Alcohol in past month**	35	25	44	41
**No regular physician**	15	14	14	22
**No religious attendance**	33	24	38	47
**Systolic BP ≥ 140 mmHg**	49	47	50	57
**BMI ≥ 25 kg/m^2^**	54	51	61	43
**No physical activity**	79	79	81	72
**HDL < 40 mg/dL**	27	23	31	29

**Table 2:** Adjusted hazards ratios* for death for smoking, score on a short index of cognitive function (SICF) in persons aged 60 years and over.

	HR (95% CL)			
	All	Age 60–69	Age 70–79	Age 80+
**Smoking status**				
**Current**	2.13 (1.75–2.59)**	3.09 (2.08–4.59)**	1.70 (1.29–2.24)**	1.39 (0.99–1.97)
**Former**	1.45 (1.25–1.65)**	1.80 (1.27–2.54)**	1.48 (1.22–1.70)**	1.16 (0.95–1.42)
**Never**	1.00	1.00	1.00	1.00
**SICF**				
**17**	0.68 (0.53–0.88)**	0.65 (0.39–1.07)	0.69 (0.50–0.97)+	0.58 (0.41–0.81)**
**14–16**	0.74 (0.61–0.92)**	0.54 (0.35–0.71)**	0.90 (0.60–1.34)	0.77 (0.65–0.92)**
**12–13**	0.73 (0.63–0.85)**	0.50 (0.36–0.71)**	0.80 (0.62–1.02)	0.95 (0.78–1.16)
**0–11**	1.00	1.00	1.00	1.00
**N**	4,915	2,102	1,633	1,180

* adjusted for age, gender, ethnicity, education;

** p < 0.01;

+ p < 0.05.

**Table 3:** Adjusted odds ratios* for death for smoking and score on a short index of cognitive function (SICF) in persons aged 60 years and over.

	Age			
Variable	All	60–69	70–79	80+
**Smoking status**				
**Current**	2.98 (2.16–4.12)**	3.89 (2.51–6.01)**	1.99 (1.33–2.97)**	1.69 (0.68–4.17)
**Former**	1.68 (1.39–2.05)**	1.90 (1.33–2.72)**	1.55 (1.20–2.00)**	2.02 (1.29–3.18)**
**Never**	1.00	1.00	1.00	1.00
**SICF**				
**17**	0.34 (0.23–0.50)**	0.33 (0.18–0.64)**	0.38 (0.24–0.59)**	0.22 (0.12–0.40)**
**14–16**	0.37 (0.27–0.52)**	0.28 (0.18–0.44)**	0.50 (0.28–0.88)+	0.34 (0.23–0.49)**
**12–13**	0.60 (0.47–0.76)**	0.46 (0.31–0.69)**	0.70 (0.50–1.00)+	0.92 (0.62–1.35)
**0–11**	1.00	1.00	1.00	1.00
**N**	4,916	2,102	1,633	1,181

* adjusted for age, gender, ethnicity, education;

** p < 0.01;

+ p < 0.05.

**Table 4:** Percentage (95% confidence interval) of never and former smokers by self report with serum cotinine ≥14 ng/mL score on a short index of cognitive function.

Stratum of cognitive function and smoking status by self report			
Lowest (score < 12)		Highest (score > 16)	
Never	Former	Never	Former
10.8 (7.3–15.6)	16.4 (10.9–23.7)	2.3 (1.1–4.6)	9.6 (6.0–15.2)

**Appendix 1:** NHANES III short index of cognitive function items (NHANES III variable name and question).

**Orientation**		
1.	HAA4D	What is today’s date?
2.	HAA5D	What is the day of the week?
3.	HAA6AD	What is your complete address: street?
4.	HAA6BD	What is your complete address: city?
5.	HAA6CD	What is your complete address: state?
6.	HAA6DD	What is your complete address: zip code?
**Immediate recall** (3 objects: apple, table, penny after 0 minutes and 5 minutes)		
7.	HAP17A1D	Apple immediate recall
8.	HAP17A2D	Table immediate recall
9.	HAP17A3D	Penny immediate recall
**Simple math** (serial subtraction of $3 from $20)		
10.	HAP18AD	First subtraction ($17)
11.	HAP18BD	Second subtraction ($14)
12.	HAP18CD	Third subtraction ($11)
13.	HAP18DD	Fourth subtraction ($7)
14.	HAP18ED	Fifth subtraction ($4)
**Delayed recall** (of three objects above)		
15.	HAP19AD	Apple
16.	HAP19BD	Table
17.	HAP19CD	Penny
SAS Program Code for Computing SICF Score*		
ARRAY Q [17] HAA4 HAA5 HAA6A HAA6B HAA6C HAA6D HAP17A1 HAP17A2 HAP17A3 HAP18AR HAP18BR HAP18CR HAP18DR HAP18ER HAP19A HAP19B HAP19C; ARRAY R [17] HAA4D HAA5D HAA6AD HAA6BD HAA6CD HAA6DD HAP17A1D HAP17A2D HAP17A3D HAP18AD HAP18BD HAP18CD HAP18DD HAP18ED HAP19AD HAP19BD HAP19CD; Do I=1 TO DIM(Q); Do I=1 to DIM(R); IF Q[I]=1 THEN R[I]=R[I] + 1; Else IF Q[I]=2 THEN R[I]=R[I] + 0; Else IF Q[I] NE 1 and Q[I] NE 2 THEN R[I]=.; END; End; Drop I;		
**SICF_SCORE=**SUM(OF HAA4D HAA5D HAA6AD HAA6BD HAA6CD HAA6DD HAP17A1D HAP17A2D HAP17A3D HAP18AD HAP18BD HAP18CD HAP18DD HAP18ED HAP19AD HAP19BD HAP19CD);		

* One point given for each correct answer to items 1–17 (Score ranges from 0 to 17).

Cronbach’s alpha = 0.82 (n = 4,916)

**Appendix 2:** Mean (se) of selected continuous characteristics by smoking status at baseline in persons aged 60 years and over (N = 4,916).

Smoking status				
	All	Never	Former	Current
**N**	4,916	2,336	1,827	753
**Cognitive function index Score**	13.66 (0.34)	13.55 (0.34)	13.85 (0.35)	13.49 (0.39)
**Age (years)**	69.90 (0.22)	71.03 (0.38)	69.64 (0.19)	67.19 (0.25)
**Systolic BP ≥ 140mmHg**	138.32 (0.44)	139.65 (0.71)	137.88 (0.61)	135.55 (1.19)
**BMI kg/m^2^**	26.99 (0.12)	27.10 (0.19)	27.45 (0.13)	25.43 (0.27)
**HDL mg/dL**	51.31 (0.42)	52.70 (0.45)	50.00 (0.71)	50.72 (0.87)
**Total cholesterol mg/dL**	223.76 (1.08)	226.78 (1.47)	221.74 (1.50)	220.13 (1.72)
